# Treatment Approaches for Altered Facial Expression: A Systematic Review in Facioscapulohumeral Muscular Dystrophy and Other Neurological Diseases

**DOI:** 10.3233/JND-230213

**Published:** 2024-04-30

**Authors:** Nathaniël B. Rasing, Willianne A. van de Geest-Buit, On Ying A. Chan, Karlien Mul, Anke Lanser, Baziel G.M. van Engelen, Corrie E. Erasmus, Agneta H. Fischer, Koen J.A.O. Ingels, Bart Post, Ietske Siemann, Jan T. Groothuis, Nicol C. Voermans

**Affiliations:** a Department of Neurology, Donders Institute for Brain, Cognition and Behaviour, Radboud University Medical Center, Nijmegen, The Netherlands; b Information Specialist, Medical Library, Radboud University, Nijmegen, The Netherlands; c Patient Representative and Chairman FSHD Advocacy Group, Patient Organization for Muscular Disease Spierziekten Nederland, Baarn, The Netherlands; d Department of Paediatric Neurology, Donders Institute for Brain, Cognition and Behaviour, Radboud University Medical Center, Amalia Children’s Hospital, Nijmegen, The Netherlands; e Department of Psychology, Social Psychology, University of Amsterdam, Amsterdam, the Netherlands; f Department of Otorhinolaryngology, Radboud University Medical Center, Nijmegen, The Netherlands; g Department of Medical Psychology, Radboud Institute for Health Sciences, Radboud University Medical Center, Nijmegen, The Netherlands; h Department of Rehabilitation, Donders Institute for Brain, Cognition and Behaviour, Radboud University Medical Center, Nijmegen, The Netherlands

**Keywords:** (MeSH terms): Muscular dystrophy, facioscapulohumeral, bell palsy, Parkinson’s disease, myotonic dystrophy, mobius syndrome, facial expression, therapeutics, psychosocial functioning

## Abstract

**Background:**

Facial weakness is a key feature of facioscapulohumeral muscular dystrophy (FSHD) and may lead to altered facial expression and subsequent psychosocial impairment. There is no cure and supportive treatments focus on optimizing physical fitness and compensation of functional disabilities.

**Objective:**

We hypothesize that symptomatic treatment options and psychosocial interventions for other neurological diseases with altered facial expression could be applicable to FSHD. Therefore, the aim of this review is to collect symptomatic treatment approaches that target facial muscle function and psychosocial interventions in various neurological diseases with altered facial expression in order to discuss the applicability to FSHD.

**Methods:**

A systematic search was performed. Selected studies had to include FSHD, Bell’s palsy, Moebius syndrome, myotonic dystrophy type 1, or Parkinson’s disease and treatment options which target altered facial expression. Data was extracted for study and patients’ characteristics, outcome assessment tools, treatment, outcome of facial expression and or psychosocial functioning.

**Results:**

Forty studies met the inclusion criteria, of which only three studies included FSHD patients exclusively. Most, twenty-one, studies were performed in patients with Bell’s palsy. Studies included twelve different therapy categories and results were assessed with different outcomes measures.

**Conclusions:**

Five therapy categories were considered applicable to FSHD: training of (non-verbal) communication compensation strategies, speech training, physical therapy, conference attendance, and smile restoration surgery. Further research is needed to establish the effect of these therapies in FSHD. We recommend to include outcome measures in these studies that cover at least cosmetic, functional, communication, and quality of life domains.

## INTRODUCTION

Facioscapulohumeral muscular dystrophy (FSHD) is a slowly progressive neuromuscular disorder. Asymmetrical weakness of muscles in the face, shoulders, and upper arms are often the first symptoms. Later in the disease course, lower limb and trunk muscles become affected [1, 2]. It is the second most common inherited muscular dystrophy in adulthood, with an estimated prevalence of 1 per 8,000 to 1 per 20,000 [3, 4]. FSHD type 1 (95% of all patients [5]) is caused by contraction of D4Z4 microsatellite repeats on chromosome 4. This contraction leads to expression of a normally repressed DUX4 gene in skeletal muscles [6]. Expression of DUX4 induces the expression of other genes, leading to apoptosis, oxidative stress, and activation of the immune system. Altogether these factors lead to muscle dystrophy and weakness [7].

**Fig. 1:**
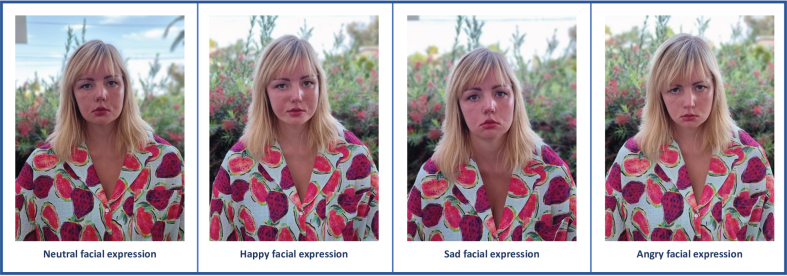
Facial expressions of a FSHD patient with facial weakness. These pictures are an example of facial expressions of a 27-year old FSHD patient with facial weakness in a neutral situation and during three primary emotions: happy, sadness and anger. Permission for usage of this pictures was granted by the patient. Adapted from ‘’Psychosocial functioning in patients with altered facial expression: a scoping review in five neurological diseases” by N.B. Rasing et al. 2023 [25].

Facial weakness is an early symptom [8] and occurs in approximately 75% of FSHD patients [9]. Three facial muscles are mostly affected: 1) zygomaticus major muscle weakness causes difficulties in raising the corners of the mouth and can lead to an altered smile; 2) orbicularis oris muscle weakness can cause asymmetry of the mouth in resting position, which is more visible when blowing the cheeks; and 3) orbicularis oculi muscle weakness leads to difficulties in closing the eyelids [1, 2]. Taken together, this can contribute to an altered facial expression or even an expressionless face [1]. Figure 1 illustrates the reduced facial expressions in a young female with FSHD.

Facial expressions are crucial in everyday communication, influencing first impressions [10], inferring emotions or personality characteristics [11], and raising empathy or involvement in others [12]. Facial expressions also elicit emotional mimicry [13], which serves as the base for smooth communication. Studies have shown that people in different cultures around the world can recognize emotions on the basis of emotional display in facial expressions [14]. Several studies have further shown that people with less or no facial expression are impaired in social interactions, because their emotions are less recognized and less mimicked. Patients with Bell’s palsy are known to experience more psychological distress [15] and have higher depression rates [16, 17], compared with people without facial weakness. Parkinson’s disease patients with facial masking are often judged by others as less attractive, supportive, social positive and more introverted, this is more pronounced in patients with more severe facial masking [18–22].

The initiative of this review arose from the observation of psychosocial distress in patients with FSHD with an altered facial expression in our recent studies [23–25]. In addition it is our clinical experience in the neuromuscular clinic that communication of FSHD patients is somewhat different compared to other patients. FSHD patients seem to be more direct, clear and redundant in their verbal communication, we hypothesized this to be the result of lack of supporting facial expression. Furthermore, an increasing number of FSHD patients at our expert centre make inquiries about available treatments. Currently, there are no curative treatments for FSHD, nor for the facial weakness it causes. Therefore, supportive treatment options will be important for reducing the negative consequences of facial weakness. We hypothesized that some supportive treatment options used in other neurological diseases with altered facial expression (Bell’s palsy, Moebius syndrome, myotonic dystrophy type 1, and Parkinson’s disease) could also be applicable to FSHD. Hence the aim of this systematic review is to provide an overview of available treatment options to improve facial expression and on therapies focussing on psychosocial consequences of having an altered facial expression in other neurological diseases with altered facial expression, and to evaluate their potential in FSHD patients.

## METHODS

A systematic literature search was performed to provide an overview of literature on symptomatic treatment options in FSHD and four other neurological diseases with altered facial expression. Each disease is presented separately in the results section. Therapies were subsequently evaluated for applicability to FSHD. Therapy efficacy, pathophysiological targets, and generalisability to FSHD patients were taken into consideration during this evaluation.

### Protocol

The methods of this systematic review were defined in advance and documented in a study protocol. The study protocol was registered at PROSPERO (CRD42020212524).

### Type of study

Included studies were randomized controlled trials, quasi randomized controlled trials, non-randomized trials, case-control studies, and case reports. The last were included, because of the expected scarcity of published literature on this subject and to minimize the chance of missing potential treatment options for FSHD patients. Only prospective study designs were included. Conference abstracts were excluded. All studies had to be published in English or Dutch. Only studies from 1990 until June 24, 2022 were included, because we expected treatment options in older studies to be outdated for improving altered facial expression.

### Participants

**Fig. 2:**
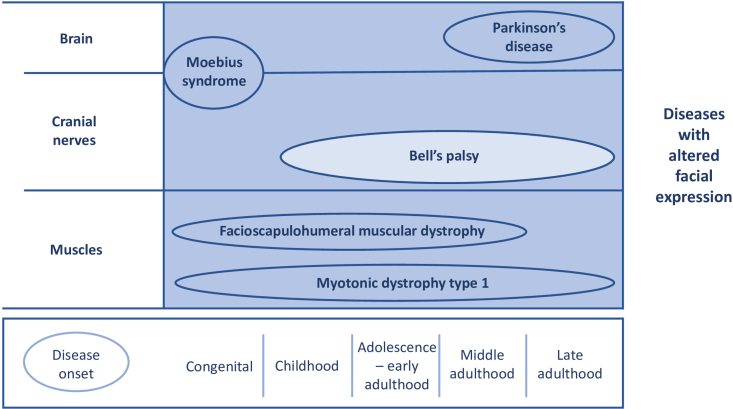
Overview for time of onset and origin of pathophysiology in the included diseases. X-axis: time of onset. Y-axis: anatomical localisation of pathophysiology. Disease progression: dark colour represents a gradual onset; light colour represents an acute onset. *Bell*’*s palsy* is an acute-onset unilateral lower motor neuron palsy, with idiopathic origin. Other symptoms that frequently occur are facial pain, altered facial sensations, dysgeusia, and hyperacusis [94]. *Facioscapulohumeral muscular dystrophy* is a slowly progressive, inherited neuromuscular disorder. It is caused by a repeat contraction of D4Z4 macrosatellite array on chromosome 4. Facial weakness is one of the first disease symptoms. Other symptoms include muscle weakness of the lower limbs and trunk [2]. *Moebius syndrome* is a congenital disease facial nerve palsy (uni- or bilateral) and abducens nerve palsy, which is caused by cranial nerve nuclei impairments [26]. It is a rare disease (estimated prevalence: 1/50,000) [95]. Studies suggest a higher risk of developing an autism spectrum disorder and cognitive impairment in patients with Moebius syndrome, although level of evidence is low [96]. *Myotonic dystrophy type 1* is a multisystem disorder, caused by an CTG triplet expansion in the DMPK gene. Cardinal symptoms are myotonia and muscle weakness in facial, trunk and distal limb muscles. Facial weakness leads to a specific facial appearance with tented upper lip. Other symptoms include cardiac conduction defects, respiratory insufficiency, and cataract [97]. *Parkinson*’*s disease* is a progressive neurodegenerative disease, which is caused by dopamine depletion, due to loss of dopaminergic neurons in the substantia nigra. One of the symptoms is hypomimia (caused by bradykinesia). Other cardinal symptoms include postural instability, rigidity, and resting tremor [98]. Adapted from ‘’Psychosocial functioning in patients with altered facial expression: a scoping review in five neurological diseases” by N.B. Rasing et al. 2023 [25].

Studies had to include patients with one of the following neurological diseases: FSHD, Bell’s palsy, Moebius syndrome, myotonic dystrophy type 1, or Parkinson’s disease. Although the clinical features in these disorders are different, all have some degree of altered facial expression or will develop this in the course of their disease (see Fig. 2). We decided to exclude studies which included multiple causes of facial paresis. By doing so, influences of other disease mechanisms were diminished. In Moebius syndrome, patients have congenital uni- or bilateral facial and abducens nerve palsy due to cranial nerve impairments [26]. Hence, facial weakness is a distinct symptom in this disease. Furthermore, studies with patients with myotonic dystrophy type 1, also known as Steinert’s disease, were included. Studies with myotonic dystrophy type 2 were excluded, since facial weakness is not a pronounced characteristic in this subgroup [27]. Lastly, studies focussing on treatment of hypomimia in Parkinson’s disease were included. Parkinson’s disease patients with hypomimia have an altered facial expression and was therefore chosen as disease of interest.

### Interventions

Studies had to focus on treatment options for altered facial expression or on improving psychosocial consequences of having an altered facial expression. Studies on treatments specifically addressing the underlying pathophysiology of other disorders than FSHD were not part of the scope of this review. For example, studies on antiviral medication or prednisolone for Bell’s palsy, specifically used in the acute stage of the disease, were excluded. Studies with these drugs could only be included when used as usual care and not as intervention. Studies on botulinum toxin treatment for synkinesis were not considered useful since they do not focus on facial weakness. Thirdly, studies with surgery or non-invasive treatments (laser therapy) involving only nerve transposition, reinnervation or nerve decompression were excluded, because these are not applicable to neuromuscular diseases in which the disease affects the muscle and not the nerve. Furthermore, studies with Parkinson’s disease medication or deep brain stimulation as intervention were excluded, because these treatment options are specific for Parkinson’s disease and not applicable to FSHD. Lastly, all studies focussing on complementary medicine (such as herbal medicine) were excluded. An exception was made for acupuncture, because this is already partly implemented in usual care around the world.

### Outcomes measures

The main focus of outcome measures were the difference of facial expressions and quality of life before and after treatment of interest.

### Search strategy

The following databases were searched: the Cochrane Library (1990 to June 24, 2020), Embase (1990 to June 24, 2020), and PubMed (1990 to June 24, 2020). The search was updated on June 24, 2022. The search strategy for PubMed is described in Table 1. The search strategies for the Cochrane Library and Embase are available upon request. In addition to the above-described search strategy, the references of included articles and articles citing one of the included articles, were also assessed for possible inclusion.

**Table 1:** PubMed search strategy (1990 to June 24, 2022)

Search	Results
#***1: Diseases***
myotonic dystrophy[MeSH Terms] OR parkinson disease[MeSH Terms] OR myotonic dystroph^ *^[Title/Abstract] OR parkinson^ *^[Title/Abstract] OR steinert disease[Title/Abstract] OR steinert’s disease[Title/Abstract] OR steinert myopath^ *^[Title/Abstract] OR steinert’s myopath^ *^[Title/Abstract] OR bell palsy[MeSH Terms] OR mobius syndrome[MeSH Terms] OR bells pals^ *^[Title/Abstract] OR mobius syndrome[Title/Abstract] OR moebius syndrome[Title/Abstract] OR muscular dystrophy, facioscapulohumeral[MeSH Terms] OR fshd^ *^[Title/Abstract] OR facioscapulohumeral muscular dystrophy[Title/Abstract] OR bell pals^ *^[Title/Abstract] OR bell’s pals^ *^[Title/Abstract]	156,263
#***2: Altered facial expression***
facial expression[MeSH Terms] OR facial paralysis[MeSH Terms] OR facial weakness[Title/Abstract] OR facial expression[Title/Abstract] OR facial paralysis[Title/Abstract] OR hypomimia[Title/Abstract] OR facial muscles[MeSH Terms] OR mimetic muscle[Title/Abstract] OR facial paresis[Title/Abstract] OR facial muscl^ *^[Title/Abstract] OR facial nerve paralysis[Title/Abstract]	41,635
#***3: Therapies***
therapy [Subheading] OR Therapeutics[MeSH Terms] OR therap^ *^[Title/Abstract] OR surgery[subheading] OR “Surgical Procedures, Operative”[Mesh] OR surg^ *^[Title/Abstract] OR mime^ *^[Title/Abstract] OR treatment^ *^[Title/Abstract] OR physiotherap^ *^[Title/Abstract] OR mirror therap^ *^[Title/Abstract] OR psycholog^ *^[Title/Abstract] OR botulin^ *^[Title/Abstract] OR botox[Title/Abstract] OR “Botulinum Toxins, Type A”[MeSH Terms] OR psychotherapy[MeSH terms] OR psychotherap^ *^[Title/Abstract] OR acupuncture[Title/Abstract] OR rehabilitation[Title/Abstract] OR intervention^ *^[Title/Abstract]	14,403,153
#1 AND #2 AND #3	1,639
#1 AND #2 AND #3 + filter: language: English and Dutch	1,390

### Study selection

The articles were screened independently for inclusion by two assessors (N.R. and W.G.) through consecutively title and abstract screening and full text screening. Disagreements about inclusion were discussed by the assessors and the principal investigator (N.V.) until consensus was reached. Covidence [28], a web-based screening and data extraction tool, was used for management of studies during the study selection procedure.

### Quality assessment

Methodological quality was assessed using three critical appraisal tools of the Joanna Briggs Institute. To ensure that quality assessment tools were appropriate for every study, different tools were used for randomized controlled trials, quasi-experimental studies, and case reports [29, 30]. The tools consist of consecutively eight, nine and thirteen questions. Questions can be scored with “yes”, “no”, “unclear”, or “not applicable”. Every question is rewarded with one point, when the answer is “yes”. A higher total score is an indication for a lower risk of bias. Quality assessment was rated independently by two assessors (N.R. and W.G.). Disagreements about the quality assessment were discussed by the assessors and the principal investigator (N.V.) until consensus was reached. Covidence [28] was also used for management of studies during the quality assessment procedure.

### Data collection

Data were collected on study (year of publication, authors, country, type of treatment, and study design); participants characteristics (number of participants, age, disease of interest, and duration of symptoms); and on outcome measures (outcome assessment tool, outcomes for facial weakness or facial expression and, if available, psychosocial outcomes).

## RESULTS

### Results of the search

**Fig. 3:**
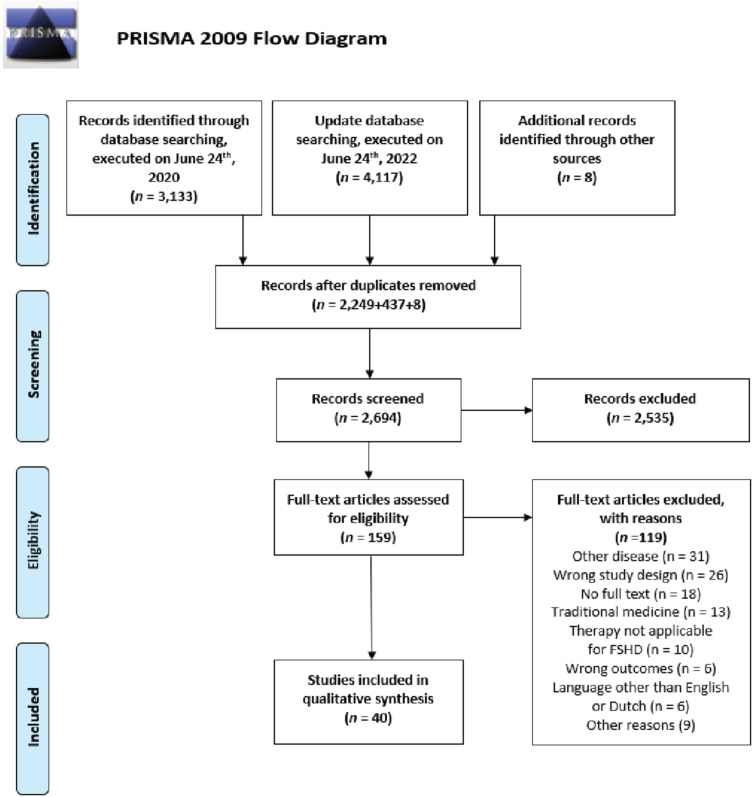
PRISMA flowchart for study inclusion. This flowchart presents the results of the literature search and the number of selected studies for each step of study inclusion.

The primary database search, executed on June 24^th^, 2020 yielded 3,133 articles (The Cochrane Library: 202, Embase: 1,709, PubMed: 1,222). A total of 2,249 articles remained after removing duplicates. Title and abstract screening resulted in 138 eligible articles for full text screening. Thirty-two articles were included after full text review. Screening of reference lists of the included studies which were cited in one of the primary included studies resulted in another eight studies for possible inclusion. Seven of these eight studies were included, and one study was excluded during title and abstract screening. Update of the search, executed on June 24^th^, 2022, yielded one extra article. Altogether, 40 studies met inclusion criteria for this review (details in Fig. 3).

### Study characteristics

In the included studies, all five neurological diseases were addressed. More than half of the 40 included studies were studies with Bell’s palsy patients (21 studies) [31–51]. Second most included study population was patients with Moebius syndrome (nine studies) [52–60]. The other three diseases were present in five (Parkinson’s disease) [61–65], three (FSHD) [66–68], and two (myotonic dystrophy type 1) [69, 70] studies. Seventeen studies were set up as randomized controlled trial, twelve studies were case reports, others had a quasi-experimental study design. Most studies were performed in Asia and North America (33% each), the other studies in Europe (28%), Africa (5%), and Australia (3%). A variety of different types of therapies were present in the included studies, with physical therapy (*n* = 10), surgery (*n* = 7), and speech therapy (*n* = 7) as most included. Characteristics of the included studies are presented in Table 2.

**Table 2:** Overview of included articles

Participants		
First author Year Country	Study design Level of health care	Sample size (% male)	Mean age (years)	Time since diagnosis	Outcome assessment tool	Treatment	Outcome for facial weakness/facial expression/psychosocial functioning/speaking performance
**Facioscapulohumeral muscular dystrophy**							
Dua [66] 2019 IND	CR SHC	1 (0%)	57	17y	None	Dermal fillers: 5 cog threads on each side of the cheek + 2 ml Juvederm ultra plus XC + 2 ml Juvederm volume XC.	Follow-up after 3wk: improvements: jawbone contour, nasolabial folds, and symmetry. Mild deflation was still observed at submalar area of most affected side. Side-effect: inflammation.
Liu [67] 2019 USA	CR SHC	1 (0%)	62	10y	None	Acupuncture: 7 points: needles in place for 30 min. 13 points: needles in place for 20 min. Manipulation after 10 min. 3x/wk for 2x 5mth.	First 3mth: no improvements. After 4mth: eye closure improvement. After 5mth: mild improvement facial expression + complete eye closure. After 10mth: improvements → facial muscle movement, eyebrow frowning, eye closure, forehead wrinkle, and muscle orbicularis oris function.
Matsumoto [68] 2016 JPN	CR THC	3 (0%)	A: 27 B: 31 C: 61	NR	None	Cartilage graft + lip resection: resection of atrophic/drooping central part of lower lip. Cartilage graft from auricular cartilage. All patients had a drooping lower lip with either functional or aesthetic problems.	A: Elevation of lower lip → improvement of teeth exposure. Advancing philtrum to caudal side + bilateral upper lip to medial side → elongation philtrum + downward adjustment upper lip. B: Resection centre lower lip + graft insertion → lower lip was elevated by 7 mm. C: Lower lip elevation by 6 mm + graft insertion, second surgery → additional elevation of 3 mm.
**Bell’s palsy**							
Alakram [31] 2010 ZAF	QE SHC	Group A: 8 (63%) Group B: 8 (38%)	Group A: 38.6 (SD = 17.7) Group B: 41.4 (SD = 16.5)	Group A: *m* = 14.1d Group B: *m* = 12.5d	HBS	TENS: treatment for 4–12wk. Both groups: prednisolone 2 mg/kg/day (max 2wk), 1x/w: hot packs (5 min.), massage (10 min.), and 10x exercises (8 in total). Exercises at home (10 repetitions). Group A: 1x/wk: additional 30 min. TENS (EV-803 Digital SD TENS). TENS: pulsed setting; freq: 12 Hz; intensity until visible twitch on paralysed side. Group B: no other treatments.	HBS recovery rates (%): Group A: 37.6 (SD = 18.1) Group B: 29.6 (SD = 12.5) Mann-Whitney tests: no significant differences between recovery rates.
Aranha [32] 2017 IND	CR THC	1 (100%)	7	3d	SB	Physiotherapy for 14d. Facial massage, proprioceptive neuromuscular facilitation (Kabat), and facial exercises.	Pre-treatment: 21; day 9 : 44; day 16 : 75.
Barbara [33] 2009 GBR	RCT THC	Group A: 9 (56%) Group B: 11 (45%)	Group A: 35 (25–58) Group B: 42 (28–56)	≤3d^1^	HBS	Physiotherapy (Kabat protocol): All patients: antiviral medication and prednisolone. Group A: Kabat neuromuscular facilitation procedure, start day 4 after onset. Group B: no additional therapy. Group C: patients from group B, who did not show clinical recovery after 2wk, did get Kabat neuromuscular facilitation procedure after day 15.	Group A: Day 4: II: *n* = 1; IV: *n* = 8; V: *n* = 2 Day 7: II: *n* = 1; III: *n* = 2; IV: *n* = 7; V: *n* = 1 Day 15: I: *n* = 2; II: *n* = 1; IV: *n* = 7; V: *n* = 1 Group B: Day 4: III: *n* = 1; IV: *n* = 7; V: *n* = 1 Day 7: II: *n* = 1; III: *n* = 4; IV: *n* = 4 Day 15: I: *n* = 2; II: *n* = 3; III: *n* = 4 Group C: 2mth: II: *n* = 5; III: *n* = 3 Only at day 15: significant difference between groups → better scores in group A.
Bokhari [34] 2010 PAK	QE SHC	49 (NR)	NR	21d to > 1y	Descriptive	Electro-acupuncture at four points: 1 session, 20–25 min/d for 10d. Then 1wk without treatment. Lastly electro-acupuncture for 5–10d. Additional methylcobalamin injection 1x/d for 5d. Majority had undergone usual care with steroids and physiotherapy, without recovery.	All patients had satisfactory recovery within 2wk. Food collection under cheek, through buccinator weakness, was completely recovered in one week. Next recovery phase was closure of eye and relief of watering of the eyes. Frowning was gradually achieved, and recovery of this symptom was chosen to be the end point for treatment. Angle of mouth was partially recovered and need long term management.
Brach [35] 1999 USA	CR THC	1 (0%)	71	14d	SB	Physiotherapy: facial neuromuscular retraining techniques (hand-held mirror or sEMG biofeedback). Before: 7 days of steroids therapy. Frequency: First 3mth: 8 sessions; month 4–7 : 4 sessions; month 8–13: 2 sessions.	Pre-treatment: 17 After 1,5mth: 41 After 7mth: 67 After 14mth: 68 Resting scores of SB were not improved (always 15), although the author observed moderate improvements.
Cayir [36] 2013 TUR	CR Unknown	1 (100%)	41	4mth	HBS	Acupuncture: 20 sessions in 2mth. Acupuncture according to Standards for Reporting Interventions in Clinical Trials of Acupuncture.	Pre-treatment: 4 Post-treatment: 2 Patient noted a 70% overall improvement.
Cederwall [37] 2006 SWE	QE THC	9 (50%)	49.8	m=8.2wk	SB Paresis index	Physiotherapy: intervention period: 26–42wk with 6–7 measurements until scores were stable. Exercise program: exercises for mouth, nose, eyes, and forehead in front of mirror; 5–10 repetitions, 2x/d (10–15 min).	All patients improved scores on SB and paresis index.^2^
Ghous [38] 2018 PAK	RCT SHC	20 (55%)^1^	38.2 (SD = 12.2)^1^	m = 2.6d (SD = 1.6)^1^	FDI	Physiotherapy Group A (*n* = 10): proprioceptive neuromuscular facilitation (Kabat protocol). Group B (*n* = 10): Kinesio taping. Both groups: Therapies: 5x/wk for 5wk; 10d steroids treatment before rehabilitation. Additional conventional physiotherapy.	FDI: statistically significant improvements on physical subscore for group A (43.3 → 92.8). No statistical improvements for group B. Pre-treatment vs post-treatment: no statistical differences in both groups for social subscore.
Manikandan [39] 2006 IND	RCT SHC	Group A: 28 (46%) Group B: 28 (39%)	Group A: 35.7 (SD = 10.4) Group B: 34,6 (SD = 13.3)	Group A: *m* = 12.5d (SD = 11.0) Group B: *m* = 11.4d (SD = 10.4)	SB	Group A (*n* = 28): facial neuromuscular re-education; tailored to patients. Group B (*n* = 28): controls; electrical stimulation (6x/wk for 2wk). Both groups: facial exercises (hand-held mirror) for 3mth, and massages.	Group A: pre-treatment: 33; post-treatment: 66* Group B: pre-treatment: 32; post-treatment: 54.5* Post treatment scores improvement was significantly higher in group A. Movement symmetry: both groups improved; significantly better in group A. Rest symmetry: both groups improved; no significant difference.
Marotta [40] 2020 ITA	RCT SHC	20 (70%)^1^	42.2 (SD = 7.6)^1^	m = 10.4mth (SD = 6.4)^1^	SB Kinovea	Group A (*n* = 10): neuromuscular electrical stimulation with shortwave diathermy therapy; electrodes on muscles: orbicularis oris, zygomaticus, and frontalis; intensity was increased until muscle contraction; 5x/wk for 4 wk. Group B (*n* = 10): sham neuromuscular electrical stimulation. Both groups: supervised re-education exercises, mime therapy, and massage.	SB: Group A: pre-treatment: 30.3; post-treatment: 47.2 Group B: pre-treatment: 29.9; post-treatment: 37.3 Subscales: Symmetry of voluntary movements: significant more improvement in group A. No significant differences in resting symmetry scores. Kinovea: More improvement in group A for symmetry ratio voluntary movement of m. zygomaticus (Group A: 81% → 94% vs group B: 77% → 82%).
Martineau [51] 2022 CAN	RCT THC	Group A: 20 (50%) Group B: 20 (60%	Group A: 47.9 (SD = 18.1) Group B: 48.2 (SD = 15.7)	<14d^1^	HBS 2.0 SB FaCE	Group A: MEPP (Mirror Effect Plus Protocol): mirror therapy (executing facial movements while looking in a mirror) for 15 min. 2x/d until recovery (HBS 2 or less). Group B: no therapy. Instructions: avoid use of excessive facial movements.	HBS: Group A: pre-treatment: 19.7; 1–3mth after onset: 7.9; 4–6mth after onset: 6.1; 1y after onset: 5.1^ *^ Group B: pre-treatment: 19.2; 1–3mth after onset: 9.5; 4–6mth after onset: 7.6; 1y after onset: 7.4^ *^ SB: Group A: pre-treatment: 30.8; 1–3mth after onset: 79.8; 4–6mth after onset: 88.9; 1y after onset: 93.9 Group B: pre-treatment: 28.0; 1–3mth after onset: 72.5; 4–6mth after onset: 82.4; 1y after onset: 83.8 FaCE: pre-treatment: no significant differences between groups. Significant better scores for group A at assessments until end of follow-up (1y after onset).
Monini [41] 2016 ITA	RCT THC	Group A: 66 (NR) Group B: 38 (NR)	Group A: 54.5 Group B: 54.5	NR	HBS FaCE	Group A: steroids for 16d. Group B: Kabat physical rehabilitation + steroids for 16d. FaCE-scale only performed in group B.	HBS: Group *A* +≤65y → *n* = 1 V to IV; *n* = 4: V to III; *n* = 4: IV to III; *n* = 28: IV to II; *n* = 6: IV to I Group *A* + >65y → *n* = 3: V to III; *n* = 6: IV to III; *n* = 13: IV to II Group *B* +≤65y → *n* = 7: V to II; *n* = 5: V to II; *n* = 16: IV to I Group *B* + >65y → *n* = 3: V to III; *n* = 3: IV to I; *n* = 4: IV to I Better recovery rates in group B (HBS: IV &V). Faster recovery in group B^ *^ (independently from age). Younger age: correlated with better recovery in both groups^ *^. FaCE: group B:≤65y: significant improvement in all subcategories;>65y: only significant improvement for facial movement, and oral function subscores.
Monini [42] 2016 ITA	RCT THC	Group A: 66 (55%) Group B: 28 (46%)	Group A: 57.1 (16–90) Group B: 55.7 (32–74)	NR	HBS	Group A: steroids, eye drops, and paracetamol. Group B: Kabat physical rehabilitation → facilitate voluntary responses of impaired muscles. Also, steroids for 10d + tapering off within 2wk.	HBS Before treatment: group A: IV: 88%, V: 12% ; group B: IV: 36%, V: 64% . Grade improvement: group A: 2; group B: 3^ *^. Odds for improving≥3 grades in group B vs A: OR = 17.73^ *^ Mean recovery speed: group B vs A: HR = 2.19^ *^
Nicastri [43] 2013 ITA	RCT Unknown	Group A: 48 (46%) Group B: 39 (56%)	Group A: 51.3 (SD = 16.1) Group B: 47.1 (16.2)	NR	HBS	Both groups: prednisone 1 mg/kg for 10d + valacyclovir 500 mg 3x/d, for 6d. Group A: Physical therapy: 45 min./session, 2x/wk for first 3mth, then 1x/wk for last 3mth + daily program. Consists of: education, massage, motion exercises (with/without mirror), strategies for drinking, eating, smiling problems, and speech training. Group B: no other treatments.	Before treatment: Group A: IV: 48%, V: 38%, VI: 15% Group B: IV: 59%, V: 21%, VI: 21% Follow-up (6mth): no statistical difference in recovery rates (≤HBS II): group A: 75%, group B: 86% . All patients with initial HBS IV were recovered. Faster recovery group A vs B (only HBS V &VI): HR = 2.24^ *^. Overall no statistical difference in time to reach recovery.
Öksüz [44] 2019 TUR	RCT THC	Group A: 20 (45%) Group B: 20 (45%)	Group A: 40.2 (SD = 16.2) Group B: 42.7 (SD = 17.0)	Group A: 3–6mth: 40% 7–12mth: 15% >12mth: 45% Group B: 3–6mth: 90% 7–12mth: 10% >12mth: 0%	HBS SB EMG	Group A: electroacupuncture+Nogier ear acupuncture, 3x/w for 4wk. Group B: no treatment. All patients (except 1): steroids therapy before study.	HBS: Group A: pre-treatment: 3.25; post-treatment: 2.30* Group B: pre-treatment: 2.95; post-treatment: 2.45* Post-treatment scores were not significantly different. SB: Group A: pre-treatment: 38.30; post-treatment: 53.10* Group B: pre-treatment: 42.35 post-treatment: 58.00* EMG: CMAP amplitude: significant improvement in group A.
Sandeep [45] 2013 IND	RCT SHC	Group A: 25 (40%) Group B: 25 (56%)	Group A: 52 (SD = 3.2) Group B: 54 (SD = 2.6)	NR	SB	Both groups: education and exercises (15x/session, 3x/d). Antiviral therapy was continued as prescribed before study inclusion. Group A: heat by infrared on affected side for 10 min. for 1wk. Electrical stimulation at facial nerve trunk (affected side), 15x/d, after 1wk, for 3wk. Intensity was increased until mild motor stimulation. Group B: no other treatments.	SB: Mean difference (pre/post-treatment): Group A: 43.1 (SD = 18.9)^ *^ Group B: 43.7 (SD = 18.3)^ *^ No statistical differences between groups.
Seffer [46] 2017 HUN	CR SHC	1 (0%)	27	26y	none	Transplantation of peripheral blood mononuclear cells and platelet-rich plasma. 25 ml blood was harvested from median cubital vein. 9.9 ml was injected in areas of facial nerve innervation of affected side. 9x/1y.	Post-treatment: improvements voluntary movements facial muscles. Facial asymmetry was reduced. Drooping angle mouth (right side) was reduced.
Starmer [47] 2015 USA	QE THC	Bell’s palsy: 22 (36%) FSHD: 3 (0%)	Bell’s palsy: 57 (33–84) FSHD: NR	NR	IOPI	Intramuscular injections with hyaluronic acid-based fillers in deficient sites until correction of air escape and interlabial gap.	Bell’s palsy: Evaluation by speech pathologist: all patients had improved articulation (plosive sounds) and less anterior spillage. Effect duration was at least 6mth. IOPI: paralyzed side: pre-treatment: 2.2kPa; post-treatment: 5.3kPa. FSHD: IOPI: pre-treatment: 0kPa (1 patients), 1kPa (2 patients); 1 patient received treatment → strength central lip: from 0 → 1kPa, left lip: 0 → 6kPa, right lip: 0 → 7kPa.
Tong [48] 2009 CHN	RCT SHC	119 (55%)^1^	12–95^1^	m=2.6d (0–12d)^1^	HBS	Group A: acupuncture: manual stimulation, 20 min/session, 3x/wk, until full recovery (max. 3mth). Group B: steroids therapy (30 mg prednisolone 2x/d + 20 mg pepcidine 2x/d, for 1wk. Group C: only usual care. All groups: usual care = eye care, home facial exercise, and education.	Improvement scores: (HBS≤3): Group A: 96.4% Group B: 86.9% Group C: 89.5% No statistical difference was found.
Tuncay [49] 2015 TUR	RCT SHC	Group A: 28 (53%) Group B: 28 (43%)	Group A: 47.7 (SD = 17.3) Group B: 41.5 (SD = 18.1)	Group A: *m* = 21.6 (SD = 12.5) Group B: *m* = 21.3d (SD = 12.0)	HBS FDI	Group A: Electrostimulation with monophasic waveform on 11 facial muscles; 3x 30 contractions, 5x/wk for 3wk. Group B: no other therapies. Both groups: steroids (60 mg/d for 10d), education for eye protection, compensation strategies, posture, and diet modification. Physical therapy: hot packs, facial massage, and facial exercises; 5x/wk for 3wk.	HBS (median rank): group A → significant better improvement. Group A: pre-treatment: 3 (2–4); post-treatment: 1 (1–3)^ *^ Group B: pre-treatment: 3 (2–4); post-treatment: 2 (1–4)^ *^ FDI physical (median rank): Group A: pre-treatment: 50; post-treatment: 100^ *^ Group B: pre-treatment: 40; post-treatment: 85^ *^ FDI social (median rank): Group A: pre-treatment: 68; post-treatment: 96^ *^ Group B: pre-treatment: 68; post-treatment: 88^ *^ Both FDI-scores: group A → significant better improvement.
Wong [50] 2008 HKG	CR SHC	1 (0%)	15	7y	HBS	Acupuncture: 10 points/session, 25x in 2mth Before acupuncture in this study: steroids for 6d, physiotherapy, tongue acupuncture (few sessions), body acupuncture for 2mth → facial asymmetry remained.	HBS: Pre-treatment: IV Post-treatment: III Patient reported: 60–70% improvement in facial muscle strength.
**Moebius syndrome**							
Amer [52] 2010 EGY	CR THC	1 (0%)	23	Not applicable (congenital disease)	Descriptive	Bilateral smile reconstruction: double staged free flap (latissimus dorsi muscle) procedure with hypoglossal and sural nerve graft. Physical therapy + mirror exercises: for 3mth.	After 4mth: first movements. At 12mth: bilateral symmetrical smile. Hemi-lingual atrophy was observed.
Bogart [53] 2016 USA	QE PHC	Group A: 22 (33%) Group B: 25 (20%) Group C: 35 (10%) Group D: 13 (19%)	Group A: 42.0 (SD = 15.3) Group B: 43.1 (SD = 16.1) Group C: 43.7 (10.7) Group D: 37.0 (7.9)	Not applicable (congenital disease)	PNSE HADS PSQ UWDSE	MSF conference 2014 Group A: Moebius patients that attended. Group B: Moebius patients that did not attend. Group C: Parents of patients with Moebius who did attend. Group D: Parents of patients with Moebius who did not attend. Participants choose themselves to attend or not attend the conference. Perceived knowledge was assessed with one open-ended question.	Moebius patients: Attenders vs non-attenders (before conference): higher companionship support, emotional support, social comfort; lower stigma; more informational support and perceived knowledge. Attenders vs non-attenders (after conference): more improvement to cope with stigma, social comfort, and perceived knowledge. No significant improvement: companionship, informational, instrumental, or emotional support, anxiety, depression, and disability self-efficacy. Parents: Attenders vs non-attenders (before conference): Higher age, older children, education, and income. Number of earlier attended conferences: association with disability self-efficacy+perceived knowledge. Attenders vs non-attenders (after conference): no differences for social support, anxiety, depression, or stigma.
Bogart [54] 2017 USA	QE PHC	Patients: 50 (24%) Parents: 57 (19%)	Patients: 41.7 (SD = 15.7) Parents: 41.9 (SD = 10.0)	Not applicable (congenital disease)	Online survey	Online survey about attending in MSF conference in 2014; examining the benefits, limitations, and reasons for participating. Survey was 4wk for conference. Secondary analysis of data from Bogart et al. [53]	Reasons for attending: companionship, emotional and informational support. Reasons for not attending: financial reasons, health/energy limitations. Conference benefits: emotional support, information supplies, and support to others. Conference limitations: no relevant information, lack of age-appropriate activities.
Domantovsky [55] 2018 CAN	QE THC	12 (25%)	13.2 (SD = 10.6)	Not applicable (congenital disease)	FaCE SMILE	Smile reanimation: segmental gracilis muscle transfer (motor nerve to masseter muscle) SMILE measurements: preoperative, postoperative, and long-term follow up (mean time: 20y). FaCE and semi-structured interviews were only used in long-term follow-up.	FaCE: Long-term follow-up: 62.3 (SD = 12.0) Highest scores for facial comfort (86.4) and social function (69.9). Lowest score for facial movement (35.6) subscale (only scoring movements by smiling: 84.1). SMILE: Oral commissure excursion: significant improvement both postoperative as long-term. Asymmetry was minimal and did not change significantly over time. Interview: all patients: quality of life improvement, more self-confidence, improved communication, and satisfaction with facial appearance. Some patients: not fulfilled expectations → feeling/looking different.
Lifchez [56] 2005 USA	CR SHC	3 (67%)	Patient 1 : 7 Patient 2 : 12 Patient 3 : 12	Not applicable (congenital disease)	Descriptive	Smile reanimation: gracilis muscle (5x) and latissimus dorsi muscle (1x) → muscle transfer, with re-innervation by the branch of trigeminal nerve (to masseter). Postoperative therapy: smile training → mirror exercises and biofeedback.	Patient 1 : 9.5y after surgery: smiles independently, without being aware of biting during smiling. No jaw occlusion. Slight elevation of modiolus left, during biting with max. force. Patient 2 : 3y after surgery: symmetrical open-mouth smile was possible, with no jaw occlusion. Patients 3: (muscle graft: 1x gracilis + 1x latissimus dorsi): 4.5y after surgery: symmetric smile, for facial movements patients needed voluntary effort and jaw closure.
Lu [57] 2013 TWN	CR SHC	6 (50%)	22.3 (5–52)	Not applicable (congenital disease)	SES CAS	One-stage smile reconstruction: gracilis muscle (12x) and rectus femoris muscle (1x) → free functional muscle transfer, with reinnervation by bilateral spinal accessory nerves. After surgery: neck immobilisation for 3wk; after 3w: electrical stimulation; after 4mth: exercises → movement of transferred muscles + smile training.	SES: (mean score): Pre-surgery: 0.7; post-surgery: 3.4 All patients had post-surgery minimal score 2 (2 teeth or lateral incisor are visible during smiling). CAS: post-surgery: stage II: 17%, stage III: 17%, stage IV: 33%, stage V: 33% . Satisfaction score was 2.8/5. All patients could synchronously smile after surgery.
Michael [58] 2015 DNK	QE Unknown	Patients: 5 (40%) Controls: 10 (50%)	Patients: 16.2 (SD = 1.92) Controls: 17.7 (SD = 2.79)	Not applicable (congenital disease)	Descriptive	Social skill workshop: learning compensatory expressive behaviour → discussions of personal experiences, role-plays, group activities, and writing. Follow-up: 6mth. Rapport was assessed with 1–6 point scale (self-report about interactions), or 1–5 point scale (observers rated videos of interactions). Non-verbal behaviour: behavioural coders; verbal behaviour: automated extraction.	Pre-treatment: more gesturing/facial expressivity, fidgeting, and less variable speech rate in controls. Both groups: Rapport: pre-treatment vs post-treatment: self-report: no differences; observers: higher post-treatment.^ *^ Non-verbal communication: pre-treatment vs post-treatment: more gesturing, facial expressivity, head movements.^ *^ Verbal communication: More speech rate variability. Slower speech rate.^ *^ Patients vs controls: (pre/post treatment): higher gesturing, and facial expressivity. Controls vs patients: (pre/post treatment): more speech rate variability, and lower fidgeting. Social competence + social anxiety in patients: no differences during measurements. Linguistic alignment: post-treatment: decreased.
Woollard [59] 2010 GBR	CR THC	20 (55%)	18 (4–46)	Not applicable (congenital disease)	HayS SurgS	One-stage (8x) or two-stage (12x) smile reconstruction: temporalis muscle (1x), bilateral gracilis muscle (1x), or bilateral latissimus dorsi muscle (18x) → free functional muscle transfer, with reinnervation: hypoglossus nerve (1x), accessory nerve (2x), or trigeminal nerve (17x).	HayS: pre-surgery: 9; post-surgery: 2.8 SurgS: good to excellent in all patients. No differences in outcomes for one-stage or two-stage procedures.
Zuker [60] 2000 CAN	QE THC	10 (30%)	7.5 (4.5–13)	Not applicable (congenital disease)	Speech protocol	Two-stage smile reconstruction: segmental gracilis muscle → free functional muscle transfer, with reinnervation: masseteric motor nerve.	Interview: pre-surgery: oral competence problems (drooling + water spillage with drinking): 70% . Post-surgery: all patients had significant improvements (no drooling + water spillage with drinking). Speech protocol (content not specified): Pre-surgery: speech problems (bilabial insufficiency, and articulation problems): 60% . Post-surgery: Improvements for these 6 (3 symptomless). Active oral commissure movements: Pre-surgery: 30% ; Post-surgery: 100% .
**Myotonic dystrophy type 1**							
Sjögreen [70] 2010 SWE	RCT THC	Group A: 4 (50%) Group B: 4 (75%)	Group A: 15 Group B: 14	NR	Non-validated questionnaire	Group A: exercise program in first 16wk. Group B: exercise program after 16wk for 16wk. Exercise program: training with oral screen for 16 min. 5x/wk. (2x 3 min.: oral screen is pulled as much, without it going out the mouth, then 5 sec pause; 10 min.: passive usage of oral screen → experiencing nasal breathing with closed mouth.	Maximal lip force: 88% had improvements; 50% had significant improvement after treatment; 3 patients had improvements before treatment. No significant difference between treatment or no treatment groups. Lip articulation: 50% was impaired. Salvia control: patients with improvements after treatment had no salvia control problems at baseline. Eating/drinking: all but 1 had no/mild difficulties.
de Swart [69] 2006 NLD	QE THC	Patients: 30 (53%) Controls: 10 (40%)	Patients: 40.4 (SD = 12.6) Controls: 34.4 (SD = 12.6)	m=12.3y (SD = 8.2)	Non-validated questionnaire	Speech protocol: 10 minutes continuous speech; first and last test: maximum repetition rate task → producing monosyllabic sequences fast and as long as possible. Other tests include: reading out loud, phonate vowels as long as possible, recite months of the year.	After warming up (part of speech protocol) → Signs of flaccid dysarthria did not improved, nor get worse. Signs of flaccid dysarthria: 1. poorer maximal performance on sound prolongation tasks. 2. Maximum repetition tasks with more variability. 3. Low maximum speech rate.
**Parkinson’s disease**							
Bryans [61] 2020 USA	QE THC	25 (96%)	69.2 (SD = 9.0)	m=5.0y (SD = 5.1)	CES CPIB VHI-30	LSVT by speech language pathologists. No specifications about details therapy, or frequency. Acoustic measures were collected and evaluated by treating therapist. CES: both patients as partner/spouse/family filled in this tool.	Pre/post treatment differences: Vocal intensity: significantly improved. Mean differences: pre-treatment to follow-up (3-6mth): CES (patient): 3.72 (1.16, 6.28)^ *^ CES (partner): 2.88 (–4.70, 10.45) Strong association between CES (patient) and CES (partner): *r* = 0.744.^ *^ CPIB: 5.34 (1.31, 9.37)^ *^ VHI-30: –11.07 (–18.99, –3.14)^ *^
Dumer [62] 2014 USA	RCT SHC	Group A: 12 (67%) Group B: 16 (75%) Group C: 17 (76%) Group D: 11 (36%)	Group A: 69.3 (SD = 10.3) Group B: 68.5 (SD = 6.7) Group C: 65.7 (SD = 8.9) Group D: 61.8 (SD = 8.6)	Group A: *m* = 5.1y Group B: *m* = 5.9y Group C: *m* = 6.7y Group D: NA	FACS Happiness rating	Group A: PD patients: ARTIC → treatment program for training of orofacial-articulatory movements → goal: improving articulation with focus on vocal intensity; 60 min./session, 4x/wk, for 4wk + daily tasks. Group B: PD patients: LSVT → treatment program for training in speaking → goal: recalibration of perception of normal loudness + adequate vocal effort. 60 min./session, 4x/wk, for 4wk + daily tasks. Group C: PD patients: no treatment. Group D: controls. Facial movements elicitation: spontaneous emotional expression tasks.	FACS: Frequency and variability higher in controls compared with PD groups.^ *^ Comparison before/after treatment: Only in group B (LSVT): improved mean scores for frequency and variability.^ *^ After treatment: frequency and variability scores: not different between group B (LSVT) and group D (controls).^ *^ Group A (ARTIC): had lower scores than group D (controls).^ *^ Happiness ratings: not different between group A (ARTIC) and B (LSVT).^ *^
Katsikitis [63] 1996 AUS	RCT SHC	Group A: 8 (75%) Group B: 8 (13%)	69.9 (SD = 5.8)^1^	m=13.5y (SD = 12.2)^1^	FACEM	Group A: Orofacial physical therapy: exercises, brushing muscles, ice applying; 1 h/session, 2x/wk, for 4wk. Group B: no treatment/contact with therapist.	FACEM: pre/post-treatment: Group A: improvements for mouth opening measure and mid-top-lip measure.^ *^ Follow-up (4wk after treatment): improvements for mouth opening measure, mid-top-lip measure, and lower-lip thickness.^ *^ Groups B: no differences post-treatment. No follow-up measures done.
Levy [64] 2020 USA	RCT THC	Group A: 19 (74%) Group B: 19 (79%) Group C: 19 (63%)	Group A: 67.9 (SD = 7.2) Group B: 67.5 (SD = 9.0) Group C: 64.2 (SD = 9.2)	Group A: *m* = 4.9y (SD = 6.9) Group B: *m* = 5.0y (SD = 5.2) Group C: *m* = 4.9y (SD = 4.2)	TA	Both treatment groups: training for greater speech output amplitude and training of sensory feedback + internal cueing; 1 h/session, 16x/mth + homework. Group A: Voice treatment: improving vocal loudness. Group B: Articulation treatment: improving articulation. Group C: no treatment.	Intelligibility assessment (TA): Group A: Pre-treatment: 53.6% Post-treatment: 85.1% ^ *^ Group B: Pre-treatment: 44.8% Post-treatment: 51.6% Group C: Pre-treatment: 64.4% Post-treatment: 52.5% ^ *^ Group A → significant increasement; group B: no significant differences; group C: significant decrease. Increasement in group A is significantly different from the changes in other groups.
Sapir [65] 2007 USA	RCT	Group A: 14 (50%) Group B: 15 (53%) Group C: 14 (50%)	Group A: 68.0 (SD = 6.0) Group B: 77.6 (SD = 8.0) Group C: NR	Group A: *m* = 9.1y (SD = 7.0) Group B: *m* = 6.3y (SD = 2.2) Group C: NA	Formant frequency Vowel goodness ratings	Group A: PD patients: LSVT → treatment program for training in speaking → exercises with maximum high and low-pitch phonation + speech exercises with increasing loudness. 50–60 min./session, 4x/wk, for 4wk. Group B: PD patients: no treatment. Group C: controls (age-matched, without neurological disease). Measurements of first (F1) and second (F2) formants from different vowels (i, u, a). Vowel rating by trained raters.	Group A: Vocal sound pressure level, F2u, F2i/F2u, and vocal goodness rating: improvements^ *^. No improvements for all other variables: F1a, F1i, F1u, F2a, and F2i. Group B and C: no improvements.

^1^Data about differences between groups was missing. ^2^Data was only presented in a figure. Precise data was not presented. ^ *^=statistical significance. Abbreviations: ARTIC = articulation treatment, CR = case report, *d* = days, EMG = electromyography, ENoG = electroneurography, freq = frequency, GaAlAs = gallium-aluminum-arsenide, *h* = hours, HR = hazard ratio, Hz = hertz, LSVT = Lee Silverman Voice Treatment, max = maximum, *m* = mean, min = minutes, MSF = Moebius Syndrome Foundation, mth = months, NA = not applicable, NR = not reported, OR = odds ratio, PD = Parkinson’s disease, QE = Quasi experimental, RCT = randomized controlled trial, sec = seconds, wk = weeks, *y* = years. Outcome assessment tools used in any of the included studies: CAS = Chuang cortical adaptation stage: staging system for functional progress of transferred muscles. Stages range from: I (no movements) to V (spontaneous smile, without unvoluntary movements. CES = Communicative Effectiveness Survey: 8 item instrument for assessing communicative effectiveness. Scores range from 0 to 32. Higher scores are an indication for better communicative effectiveness. CPIB = Communicative Participation Item Bank: 10 questions for assessing communicative participation. Every question is score with a 4-point scale. Scores are converted into T-scores. Higher scores are more favourable. FaCE = facial clinimetric evaluation scale: 15 item instrument with six domains covering facial functioning. Total score ranges from 0–100. Higher scores are an indication for better facial function. FACEM = facial expression measurement: program with model of the face with 12 facial measures. FACS = Facial action coding system: anatomically based system for assessing facial expressions and movements. FDI = Facial disability index (FDI): consists of two domains, namely physical function, and social/well-being function. Scores are: 0–100, with 100 as best score. HADS = Hospital Anxiety and Depression Scale: 14 item instrument for assessing anxiety or depression. Scores range from 0–21 per subscale. Higher scores give a greater indication for anxiety, or depression. Happiness rating: Rating used for happiness indication. Scores range from 1 (not very happy) to 7 (extremely happy). Hay’s rating scale = Assessment tool for static position and active movements improvements after surgery. Scores range from 1 (perfect) to 9 (very marked imperfection). HBS = House-Brackmann scale. This is a facial grading system, ranging from I (normal function) to VI (total facial paralysis). HBS 2.0 = updated version of House-Brackmann scale. This facial grading system ranges from 4 (normal function) to 24 (total facial paralysis). Points are given per facial region: Eye, eyebrow, nasolabial fold, and oral region. IOPI = Iowa Oral Performance Instrument: device for measuring lip pressure in kilopascals. Paresis index: assessment tool for evaluating facial paresis in resting position and during voluntary movements. Scores range from 0–10, with higher scores as an indication for more facial paresis. PNSE = Positive and Negative Social Exchange: tool for assessing social support with 4 subscales: companionship, emotional, informational, and instrumental. Scores on 5-point scale, with higher scores as indication for more social support. PSQ = Perceived Stigma Questionnaire: 21 item instrument for assessing stigma by patients with visible differences. Every question has a 5-point scale, ranging from never to always. Higher scores are an indication for more perceived stigma. Satisfaction score = non-validated questionnaire for evaluating satisfaction after smile surgery. Scores range from 0–5, with 5 as maximum satisfaction score. SB = Sunnybrook facial grading system. This is a tool for assessing symmetry of facial movements and resting symmetry. Scores range from 0 (total facial paralysis) to 100 (normal symmetry). SES = Smile excursion score: scores the visibility of teeth during a wide smile. Scores range from 0 (no teeth visible) to 4 (teeth, or premolar tooth visible). SMILE = Scaled Measurement of Improvement in Lip Excursion: for assessing facial function by using iris diameter as scale of reference for facial features in a photo. SurgS = Surgeon’s score: non-validated score system performed by surgeons for assessing facial asymmetry (static and dynamic). Scores are: ‘poor’, ‘good’, or ‘excellent’. TA = Transcription accuracy: percentage of correct transcribed words during a listener’s transcription. It is used as an intelligibility measurement. UWDSE = University of Washington Disability Self-Efficacy Scale: 6 item instrument for assessing self-efficacy by patients with disabilities or chronic illness. Scores range from 6–30, higher scores are an indication for less self-efficacy. VHI-30 = Voice Handicap Index: 30 item instrument for assessing impact of voice disorders in daily life. Scores range from 0–120. Higher scores are an indication for more self-perceived impact of voice disorder.

### Quality assessment

The randomized controlled trials scored on average eight out of thirteen points. Two questions about blinding were mainly not applicable to the included studies. The non-randomized controlled trials scored on average five out of eight points. Two questions were not applicable since these questions evaluated differences between included participants groups which was relevant in only three of the non-randomized controlled trials [31, 58, 69]. The other nine trials had only a single participant group. The case reports scored on average seven out of eight points. The main limitation of these studies was a lack of described medical history of the included patients, as six out of eleven studies did not report this adequately. All details of the quality assessments are shown in Tables 3–5, respectively.

**Table 3:** Quality assessments of included randomized controlled trials (Joanna Briggs Institute Critical Appraisal Checklist for Randomized Controlled Trials)

	1.	2.	3.	4.	5.	6.	7.	8.	9.	10.	11.	12.	13.	Overall
	True	Concealed	Groups at	Blinding	Blinding	Blinding	Similar	Follow-	Intention to	Same	Outcome	Statistical	Trial	score
	randomization	allocation	baseline	participants	therapist	outcome	group	up	treat	outcome	measures in	analysis	design
						assessors	treatment		analysis	measurements	reliable way
Barbara et al. 2009 [33]	Yes	NR	Yes	NA	NA	NR	Yes	Yes	Yes	Yes	No	Yes	Yes	8
Dumer et al. 2014 [62]	Yes	NR	Yes	NA	NA	Yes	No	Yes	Yes	Yes	Yes	Yes	Yes	9
Ghous et al. 2018 [38]	Yes	Yes	No	NA	NA	NR	Yes	NR	Yes	Yes	NR	No	Yes	6
Katsikitis et al. 1996 [63]	Yes	NR	No	NA	NA	No	No	Yes	Yes	Yes	Yes	No	Yes	6
Levy et al. 2020 [64]	Yes	Yes	Yes	NA	NA	Yes	Yes	Yes	Yes	Yes	Yes	Yes	Yes	11
Manikandan 2006 [39]	Yes	Yes	Yes	NA	NA	NR	No	Yes	Yes	Yes	No	Yes	Yes	8
Marotta et al. 2020 [40]	Yes	Yes	NR	Yes	NA	Yes	Yes	Yes	Yes	Yes	No	Yes	Yes	10
Martineau et al. 2022 [51]	Yes	Yes	Yes	NA	NA	Yes	No	Yes	Yes	Yes	Yes	Yes	Yes	10
Monini et al. 2016 [41]	Yes	NR	NR	NA	NA	NR	Yes	Yes	Yes	Yes	NR	Yes	Yes	7
Monini et al. 2016 [42]	No	No	No	NA	NA	No	No	Yes	Yes	No	No	Yes	No	3
Nicastri et al. 2013 [43]	Yes	Yes	Yes	NA	NA	Yes	Yes	No	Yes	Yes	No	Yes	Yes	9
Öksüz et al. 2019 [44]	Yes	Yes	No	NA	NA	Yes	Yes	Yes	Yes	Yes	No	No	Yes	8
Sapir et al. 2007 [65]	Yes	NR	Yes	NA	NA	Yes	Yes	NR	Yes	Yes	Yes	Yes	Yes	9
Sjögreen et al. 2010 [70]	No	NR	Yes	NA	NA	NR	Yes	Yes	Yes	Yes	Yes	No	Yes	7
Tong et al. 2009 [48]	Yes	Yes	NR	NA	NA	Yes	Yes	Yes	Yes	Yes	No	No	Yes	8
Tuncay et al. 2015 [49]	Yes	Yes	Yes	NA	NA	Yes	Yes	No	Yes	Yes	Yes	Yes	Yes	10

NA=not applicable, NR = not reported. Questions used in the Joanna Briggs Institute Critical Appraisal Checklist for Randomized Controlled Trials: 1: Was true randomization used for assignment of participants to treatment groups?. 2: Was allocation to treatment groups concealed?. 3: Were treatment groups similar at the baseline?. 4: Were participants blind to treatment assignment?. 5: Were those delivering treatment blind to treatment assignment?. 6: Were outcomes assessors blind to treatment assignment?. 7: Were treatment groups treated identically other than the intervention of interest?. 8: Was follow up complete and if not, were differences between groups in terms of their follow up adequately described and analyzed?. 9: Were participants analyzed in the groups to which they were randomized?. 10: Were outcomes measured in the same way for treatment groups?. 11: Were outcomes measured in a reliable way?. 12: Was appropriate statistical analysis used?. 13: Was the trial design appropriate, and any deviations from the standard RCT design (individual randomization, parallel groups) accounted for in the conduct and analysis of the trial?.

**Table 4:** Quality assessments of included non-randomized controlled trials (Joanna Briggs Institute Critical Appraisal Checklist for Quasi-Experimental Studies)

	1.	2.	3.	4.	5.	6.	7.	8.	9.	Overall
	Cause and	Groups at	Similar	Control	Multiple	Follow-	Outcome measurements	Outcome measurements	Statistical	score
	effect	baseline	group	group	outcome	up	in the same way	in reliable way	analysis
			treatment		measurements
Alakram et al. 2010 [31]	Yes	No	Yes	Yes	No	No	Yes	No	Yes	5
Bogart et al. 2016 [53]	Yes	NA	NA	No	Yes	Yes	NA	Yes	Yes	5
Bogart et al. 2017 [54]	Yes	NA	NA	No	No	NA	NA	Yes	NA	2
Bokhari et al. 2010 [34]	Yes	NA	NA	No	No	Yes	NR	No	No	2
Bryans et al. 2020 [61]	Yes	NA	NA	No	Yes	Yes	NA	Yes	Yes	5
Cederwall et al. 2006 [37]	Yes	NA	NA	No	Yes	Yes	NA	Yes	Yes	5
Domantovsky et al. 2018 [55]	Yes	NA	NA	No	Yes	Yes	NA	No	Yes	4
Michael et al. 2015 [58]	Yes	Yes	Yes	Yes	Yes	Yes	Yes	Yes	Yes	9
Starmer et al. 2015 [47]	Yes	NA	NA	No	Yes	Yes	NA	Yes	Yes	5
de Swart et al. 2006 [69]	Yes	Yes	Yes	Yes	Yes	NA	Yes	Yes	Yes	8
Woollard et al. 2010 [59]	Yes	NA	NA	No	Yes	Yes	NA	Yes	Yes	5
Zuker et al. 2000 [60]	Yes	NA	NA	No	Yes	Yes	NA	No	Yes	4

NA=not applicable, NR = not reported. Questions used in the Joanna Briggs Institute Critical Appraisal Checklist for Quasi-Experimental Studies: 1: Is it clear in the study what is the ‘cause’ and what is the ‘effect’ (i.e. there is no confusion about which variable comes first)?. 2: Were the participants included in any comparisons similar?. 3: Were the participants included in any comparisons receiving similar treatment/care, other than the exposure or intervention of interest?. 4: Was there a control group?. 5: Were there multiple measurements of the outcome both pre and post the intervention/exposure?. 6: Was follow up complete and if not, were differences between groups in terms of their follow up adequately described and analyzed?. 7: Were the outcomes of participants included in any comparisons measured in the same way?. 8: Were outcomes measured in a reliable way?. 9: Was appropriate statistical analysis used?.

**Table 5:** Quality assessments of included case reports (Joanna Briggs Institute Critical Appraisal Checklist for Case Reports)

	1.	2.	3.	4.	5.	6.	7.	8.	Overall
	Patient’s	Patient’s	Current	Diagnostic tests,	Intervention	Post-intervention	Adverse	Takeaway	score
	demographics	history	clinical	assessment methods,		condition	events	lesson
			condition	and results
Amer et al. 2010 [52]	Yes	No	Yes	No	Yes	Yes	Yes	Yes	6
Aranha et al. 2017 [32]	Yes	No	Yes	Yes	Yes	Yes	No	Yes	6
Brach et al. 1999 [35]	Yes	No	Yes	Yes	Yes	Yes	Yes	Yes	7
Cayir et al. 2013 [36]	Yes	No	Yes	Yes	Yes	Yes	Yes	Yes	7
Dua et al. 2019 [66]	Yes	No	Yes	Yes	Yes	Yes	Yes	Yes	7
Lifchez et al. 2005 [56]	Yes	Yes	Yes	Yes	Yes	Yes	Yes	Yes	8
Liu et al. 2019 [67]	Yes	Yes	Yes	No	Yes	Yes	No	Yes	6
Lu et al. 2013 [57]	Yes	Yes	Yes	Yes	Yes	Yes	Yes	Yes	8
Matsumoto et al. 2016 [68]	Yes	No	Yes	No	Yes	Yes	No	Yes	5
Seffer et al. 2017 [46]	Yes	Yes	Yes	Yes	Yes	Yes	Yes	Yes	8
Wong et al. 2008 [50]	Yes	Yes	Yes	Yes	Yes	Yes	Yes	Yes	8

NA=not applicable, NR = not reported. Questions used in the Joanna Briggs Institute Critical Appraisal Checklist for Case Reports: 1: Were patient’s demographic characteristics clearly described?. 2: Was the patient’s history clearly described and presented as a timeline?. 3: Was the current clinical condition of the patient on presentation clearly described?. 4: Were diagnostic tests or assessment methods and the results clearly described?. 5: Was the intervention(s) or treatment procedure(s) clearly described?. 6: Was the post-intervention clinical condition clearly described?. 7: Were adverse events (harms) or unanticipated events identified and described?. 8: Does the case report provide takeaway lessons?.

### Study outcomes

Study outcomes are presented per neurological disease. An overview of all outcomes measures is shown in Table 2. We discuss the main findings below.

### FSHD

Only three case reports with FSHD patients were included. The studies did not use any of the universally used assessment tools or other systematic outcome measurements. In Dua et al. [66] a 57 year old woman received dermal fillers with cog threads on both sides of the cheek. After three weeks, the patient noted improvements of jawbone contour, nasolabial folds, and facial symmetry, but there was still a deflation at the most affected side. Acupuncture was the treatment of interest in Liu et al. [67], which was given in two groups of acupuncture points to a 62-year-old woman for two periods of five months. After ten months, improvements were observed by herself and her practitioner for several facial muscle functions, such as eye closure and top lip movements. The last included study on FSHD by Matsumoto et al. [68] performed surgery for patients with functional and aesthetic problems because of a drooping lower lip, due to facial weakness and muscle atrophy. The surgery procedure consisted of introducing auricular cartilage craft in the lower lip, to ensure lower lip elevation. All three included patients had a lower lip elevation of minimal four to seven millimetres. Only technical surgical outcomes were given as outcome measure.

### Bell’s palsy

Twenty-one studies were included with Bell’s palsy patients with five different treatment options, most frequently physical therapy. Mean time since diagnosis varied from less than one day [31] to 26 years [46]. Most used outcome measurement scores were House-Brackmann scale (HBS) and Sunnybrook facial grading system (SB), with respectively eleven [31, 33, 36, 41–44, 48–51] and eight [32, 35, 37, 39, 40, 44, 45, 51] studies. HBS is a scale for assessing facial paresis with scores range from I (normal function) to VI (complete facial paralysis). SB is a tool for assessing (a)symmetry of facial movements and synkinesis with the scores range from 0 (complete facial paralysis) to 100 (normal symmetry).

Outcomes of studies with physical therapy varied, regarding recovery rate and time to recovery, although most studies showed better outcomes for physical therapy, compared to usual care. Out of the nine studies with physical therapy, five contained Kabat physical therapy [32, 33, 38, 41, 42]. This is a proprioceptive neuromuscular facilitation method originating from Kabat et al. [71]. Two studies of Monini [41, 42] showed that some patients who received Kabat physical therapy had more improvements on HBS and a faster recovery, compared with controls who received usual care. Patients with HBS IV or V, who received Kabat physical therapy, had better recovery rates than controls and other patients. Furthermore, patients in the therapy group had on average a faster recovery [41]. Similar results were seen in another study of Monini [42], were patients who received Kabat physical therapy had 17 times the odds for improving≥3 grades on HBS score (odds ratio = 17.73), compared with a control group. Barbara et al. [33], a study which looked also at Kabat physical therapy, showed significant better improvements in HBS for therapy group, at day 15 after start of treatment. Similar results were seen in Ghous et al. [38], where patients who received Kabat physical therapy had statistical improvements on the physical subscore of a facial disability assessment tool (FDI). Social subscores of FDI failed significance.

Manikandan [39] also showed significantly more improvement for the physical therapy group (facial neuromuscular re-education) compared with control group, although both groups improved over time. Cederwall et al. [37] performed a study with a quasi-experimental study design, showing that 26 to 42 weeks of physical therapy resulted in improvements in the SB scale. A control group was lacking and data were only presented graphically. In Martineau et al. [51] intervention method was mirror therapy. Patients did twice a day facial exercises in front of a mirror. There were significant better outcomes for the therapy group compared with the control group for HBS scale and FaCE (a facial clinimetric evaluation scale) outcomes during follow-up. There were no significant differences for SB scale outcomes.

In contrast to earlier mentioned studies, Nicastri et al. [43] found no differences in recovery rate and time to recovery between the physical therapy and control group. Only more severely affected patients (HBS V and VI) had faster recovery after physical therapy.

Two case reports showed improvements in facial symmetry after physical therapy. Aranha et al. [32] reported about a seven-year-old patient and showed improvements in the SB scale (from 21 to 75 points) after 14 days of Kabat physical therapy. Another case report by Brach et al. [35] about a 71-year-old, also reported an increase in SB scale (from 17 to 68 points) after physical therapy. In this case report the physical therapy consisted of facial neuromuscular retraining techniques and duration of follow up was 14 months.

Four studies investigated the effects of electrical stimulation on facial palsy. Three of these studies found no significant difference for recovery rates between the therapy and control group. Alakram et al. [31] found no statistical differences in recovery rates (37.6% vs 29.6%) in HBS scores between the group treated with transcutaneous electrical nerve stimulation and the control group. Marotta et al. [40] found no differences between neuromuscular electrical stimulation and control group as well. SB scores, respectively 47.2 and 37.3, did not statistically differ after treatment. However, they did find more symmetry in voluntary movements in the therapy group. Sandeep et al. [45] added heat by infrared to electrical stimulation. In this study, no statistical difference after treatment between therapy and control groups (mean difference in SB scores was respectively 43.1 and 43.7) was reported. In contrast to the other three studies, Tuncay et al. [49] did find significant more improvement in both facial grading (mean HBS: III to I) and facial disability (FDI) for the therapy group, compared to controls.

Outcomes after acupuncture as treatment for improving facial palsy differed. Three studies, of which two case reports, showed improvements after acupuncture, [34, 36, 50] and two randomized controlled trials did not find statistically significant improvements [44, 48]. One of the randomized controlled trials (Öksüz et al. [44]) reviewed acupuncture treatment (19 selected points in a meridian) in patients with a disease duration of minimal three months. The other randomized controlled trial (Tong et al. [48]) included patients with a disease duration of maximum 12 days and used eight points in a meridian for acupuncture. Post-treatment there were no statistically differences between acupuncture and control groups for facial grading scores (HBS) in both randomized controlled trials [44, 48]. However Öksüz et al. [44] did find improvements in electromyography values favouring the acupuncture group. Two case reports showed improvements in facial grading (HBS) after acupuncture. Cayir et al. [36] reported an improvement of HBS IV to II after 20 sessions (15 selected points in a meridian), and Wong et al. [50] showed an improvement of HBS IV to III after 25 sessions (10 selected points per session). A Pakistani study by Bokhari et al. [34] also reported improvements after acupuncture (seven selected points in a meridian), although only descriptive results were shown and none of the widely used outcome assessment tools were used.

The following two treatment options were only included in one study each: blood injection and intramuscular injections with hyaluronic acid-based fillers. A case report by Seffer et al. [46] investigated the effects of subcutaneous and muscular injection of peripheral homologous blood at affected facial side. Improvements were reported in facial movements and facial symmetry, but an objective measurement was not used. Starmer et al. [47] used intramuscular injections with hyaluronic acid-based filler at affected sides. Lip pressure (Iowa Oral Performance Instrument) improved after treatment (2.2kPa to 5.3kPa) and all patients had improvements for articulation and less anterior spillage. Besides Bell’s palsy patients, one FSHD patient was also included. Lip pressure improved from 0kPa to 6kPa (right side of lip) and from 0kPa to 7kPa (left side of lip) in this patient.

### Moebius syndrome

Smile reanimation surgery techniques were the intervention method in six out of the nine included studies. All these studies had a quasi-experimental design (*n* = 2) [55, 60] or a case report design (*n* = 4) [52, 56, 57, 59]. They all performed a free functional muscle transfer with reinnervation and a nerve graft. Used muscles varied: latissimus dorsi, gracilis, rectus femoris, or temporalis muscle. All six studies reported improvements, measured with variable outcome measurements. Four studies [55–57, 60] used only, or in most patients, the gracilis muscle for the free muscle transfer. Domantovsky et al. [55] had a long-term follow-up duration of 20 years, in which they assessed facial functioning with FaCE. A mean score of 62.3 out of 100 was found during follow-up. The lowest subscore was for facial movements (35.6 out of 100). All patients reported improvements in quality of life, communication, and self-confidence. However, surgery did not fulfil expectations in all patients; in an interview they noted that they still talked, looked and felt different than others. A Canadian study by Zuker et al. [60] found improvements for all patients: 70% of the participants had oral dysfunction (drooling and fluid loss while drinking) before surgery, which significantly improved after surgery. Six patients with speech difficulties all experienced improvements after surgery, and three of these group of patients were even symptomless. Lifchez et al. [56] and Lu et al. [57] both mostly used gracilis muscle for the free muscle transfer, Lifchez et al. [56] used the latissimus dorsi muscle once and Lu et al. [57] used the rectus femoris muscle once. Lifchez et al. [56] noted that two of the three patients could smile independently of jaw closure after surgery. The other case report (Lu et al. [57]) showed smile excursion (smile excursion score) improvements after surgery, and all patients could synchronously smile after the intervention. Two other case reports mostly used the latissimus dorsi muscle for the free muscle transfer. Amer et al. [52] reported a bilateral symmetrical smile 12 months after surgery. Woollard et al. [59] noted improvements for static and active movements (Hay’s rating scale) and good to excellent facial symmetry (surgeon’s impression).

Three studies were included with other treatment options for Moebius syndrome patients, of which two articles focused on a Moebius Syndrome Foundation conference in 2014. Bogart et al. (2016) [53] investigated the differences in social and emotional outcomes between attenders and non-attenders. Attenders tended to have more improvements for coping with stigma, social comfort, and perceived knowledge than non-attenders. There were no differences between attenders and non-attenders for companionship, anxiety, depression, and disability self-efficacy. Bogart et al. (2017) [54] looked at the reasons for attending or not attending the conference, using data from Bogart et al. (2016) [53]. Most frequently mentioned reasons for attending were: getting companionship, emotional support, and informational support. The most common reasons for not attending were financial reasons and energy limitations. Conference limitations were also assessed and most mentioned were, lack of age-appropriate activities and lack of relevant information.

The last included study with Moebius syndrome patients is a Danish study by Michael et al. [58], investigating the benefits of a social skill workshop for learning compensatory expressive behaviour. Both a group with Moebius syndrome and a group with controls (people without Moebius syndrome) were included and observed during social interactions. The social workshop consisted of group discussions, role-plays, group activities, and writings sessions. Comparisons between both groups before treatment, showed more facial expressivity, more fidgeting, more gesturing, and less variable speech rate in the control group. Both verbal and nonverbal communication improved after the workshop: more gesturing, more facial expressivity and more head movements were observed in both patients and controls after the workshop. Although both groups improved, the increase in gesture rates, facial expressivity, and fidgeting was statistically higher in patients. Moreover, speech rate variability increased, but speech rate was lower in both groups after the workshop. Rapport (‘the ability to connect with others in a way that creates a climate of thrust and understanding’) [72] was not increased when measured with self-reports, but observers did report a significant increase in both groups. Social anxiety and social competence assessments in the Moebius syndrome group did not differ after treatment.

### Myotonic dystrophy type 1

The two included studies with myotonic dystrophy type 1 patients focused on respectively the influence of warming up on speech quality [69], and on an exercise program for improving lip muscle strength [70]. De Swart et al. [69] made a ten-minute speech protocol to review the effect of warming up on myotonia and on flaccid dysarthria (due to facial weakness), because of the aim of this review focus was only on outcomes referring to the flaccid dysarthria. Signs of flaccid dysarthria were still visible after warming up: maximal performance on sound prolongation tasks was poor, more variability on maximum repetition rate tasks, and maximum speech rate was low. However, warming up did not worsen the symptoms of flaccid dysarthria. Sjögreen et al. [70] found that a 16-week exercise program with an oral screen led to lip-strength improvements in 88% of the patients (*n* = 7). There was no statistical difference with patients who did not receive treatment.

### Parkinson’s disease

The studies on Parkinson’s disease used voice treatment or orofacial physical therapy. The outcomes after treatment were similar in these studies, as they all reported some improvement. Every study used different outcome assessment methods.

Lee Silverman voice treatment (LSVT) was used in three studies [61, 62, 65]. This is a communication intervention for improving vocal intensity in Parkinson’s disease patients, by exercises varying from saying words to conversational speech [61]. Bryans et al. [61] reported a significantly improvement of vocal intensity after LSVT. Besides, patients noted improvement in effectiveness of their speech in daily conversations (Communicative Effectiveness Survey). Dumer et al. [62] reviewed not only LSVT, but also ARTIC, wherein orofacial movements are trained to improve articulation. Assessments for facial expression movements (FACS), showed only improvements for frequency and variability in the LSVT group. After treatment, there were no longer statistical differences for frequency and variability for facial expressions between controls and LSVT group. This is in contrast with before the LSVT sessions, as frequency and variability for facial expressions were lower in Parkinson’s disease patients. A study by Sapir et al. [65] investigated the influence of LSVT on formant frequency and vowel goodness ratings (vowels produced in a sentence). They reported improvements in some formants and in vowel goodness ratings.

The only included study from Australia by Katsikitis et al. [63], noted improvements for mouth opening, after orofacial physical therapy, which contained exercises and brushing of muscles. This improvement was not seen in the control group. Lastly, Levy et al. [64] compared voice treatment with articulation treatment and results were presented with intelligibility assessment (TA). There was only a significant increasement after treatment in the voice treatment group, as the percentage of correct transcribed words by listeners increased from 53.6% to 85.1% .

## DISCUSSION

The aim of this systematic review was to provide an overview of research on available treatments for improving facial muscle function and on therapies focussing on psychosocial consequences of having an altered facial expression in other neurological diseases to evaluate their potential in FSHD patients. We encountered only limited overlap for therapies between the different neurological diseases. A total of forty studies were included with twelve different treatment categories, of which acupuncture, physical therapy, speech treatment, and surgery were the intervention of interest in at least two different neurological diseases. The studies on FSHD were not performed in a systematic manner. Based on the results in the other neurological diseases, we consider several interventions of interest for FSHD: teaching (non-verbal) communication compensation strategies, speech training, physical therapy, conference attendance, and smile restoration surgery. The approaches call for systematic prospective research. The main findings are discussed below.

### Research gap and recommendations

This systematic review was set up because of observations of psychosocial distress in FSHD patients with altered facial expression and lack of symptomatic treatment options. This scarcity was reflected by the low number of studies in FSHD in this review (*n* = 3). All three studies had a case-report design and lacked systematic outcome measures. Hence, level of evidence is low.

*Compensation strategies.* We consider teaching compensation strategies a potentially beneficial treatment option for FSHD patients. Patients with altered facial expression develop compensation strategies over time [73–75]. Compensation strategies can also be successfully taught, as Michael et al. [58] reported improvements in both verbal and non-verbal communication for Moebius syndrome patients. In a recent questionnaire study and qualitative study, we showed that younger FSHD patients experience more psychosocial distress because of facial weakness than older patients [23, 24]. Therefore, young FSHD patients could particularly benefit from learning compensation strategies. In addition to learning compensation strategies during a social skill workshop, other training modes could be considered: information provided by online modules, 3D model interactions, applications, or through peer-support. Learning how to deal with the consequences of facial weakness and learning how to optimally inform others, could also be implemented in a social skill workshop. Acceptance and Commitment Therapy (ACT) [76], a behaviour therapy focussing on improving psychosocial flexibility, might help in this learning process. Further research could investigate if FSHD patients benefit from learning compensation strategies and if combining this with ACT gives additional improvements on psychosocial outcomes.

*Communication.* Communication difficulties are common in FSHD patients (35%) [77, 78]. Nevertheless, treatment options aiming to improve communication have not been studied for FSHD patients. This in contrast to Parkinson’s disease and myotonic dystrophy [61, 62, 64, 69]. The impairments in communication in FSHD patients could be due to speech difficulties, but the influence of social interaction due to lack of facial expression could also interfere in this impairment. Therefore, it could be beneficial to improve both aspects, for instance by implementing speech training and learning compensation strategies in one training.

*Patient conferences.* Studies with Moebius syndrome patients reported about benefits and identified reasons for (not) attending Moebius syndrome patient conferences [53, 54]. Similar studies in FSHD have not been performed. These could give insight in the perspective of FSHD patients on attending in a conference. Conferences are important for sharing patients’ perspectives of their disease, for informing patients about latest insights in research progression and it is a place to introduce treatment options. It is also a place to facilitate peer support, which is support (emotional, informational, or appraisal) by another patient with the same disease or another disease with similar consequences by sharing experiences, provide suggestions, to enhance quality of life [79]. Peer support as part of shared medical appointment in patients with neurological diseases (FSHD as second largest patient category) has shown that it can improve self-reported quality of life [80]. Furthermore, it could be beneficial to supply information on facial weakness and its psychosocial consequences to a wider audience. That is especially important as patients with altered facial expression are perceived as more negative [19, 21, 22, 81, 82] and when people are aware of the consequences of having facial weakness, prejudgements could potentially be reduced.

*Physical therapy.* Physical therapy has been incorporated in treatments of Bell’s palsy and Parkinson’s disease, but applicability to FSHD is uncertain. Most studies on Bell’s palsy patients showed tendency to improved recovery rates after physical therapy [33, 37–39, 41, 42, 51]. Previously, two reviews have performed a literature search on physical therapy in Bell’s palsy patients [83, 84]. Both reviews concluded that there was neither benefit nor harm associated with physical therapy. However, these conclusions were based on searches completed in 2011 [83] and 2012 [84]. Subsequent to these reviews, six new studies were conducted. Among these, four studies reported more improvements after physical therapy [41–43, 51]. The remaining two studies were either of low quality [38], or a case report [32]. Consequently, since the searches of Baugh et al. [84] and Texeira et al. [83], the newer performed studies on this subject suggest more favourable outcomes for the groups undergoing physical therapy. Besides Bell’s palsy, one study reported improved mouth opening after orofacial physical therapy in Parkinson’s disease [63]. Because of the different pathophysiology (see Fig. 2), prediction of the effect of physical therapy in FSHD patients is difficult. It is currently unknown to what extent dystrophic muscles are capable of adaptation and strengthening by training. Skeletal muscle strength training in FSHD tends to be safe with limited improvements [85]. Aerobic training is also a safe intervention resulting in reduced fatigue [86] and increased walking speed [87]. Applicability of physical therapy for facial muscles has never been investigated in FSHD. Research could clarify if physical therapy can help in improving facial expressions.

*Plastic surgery.* Surgery as a treatment for facial weakness has not been studied systematically. Only one case series reported the effect of surgery on facial weakness in FSHD patients [68]. All three patients had a drooping lower lip with either functional or aesthetic problems. There is another case report on facial surgery in a FSHD patient, however facial weakness was not the indication and this study was therefore not included in the review. In this article a patient with early onset FSHD had surgery to reduce macroglossia and a frontal open bite through an osteotomy of the mandible in combination with a tongue reduction. No studies were performed on smile-restoration surgery in FSHD. This technique is often used in Moebius syndrome patients, with reported improvements in all included studies [52, 55–57, 59, 60]. Smile restoration surgery could be effective in FSHD patients who are severely affected by facial weakness, since the pathophysiology of FSHD is limited to muscles and does not affect nerve function. One major limitation in smile restoration in FSHD patients is the slowly but progressive nature of the muscle dystrophy and therefore the risk of transferring a potentially affected muscle. The Labbé technique (a lengthening temporalis myoplasty) where a tendon of the temporalis muscle is replaced from the coronoid process to the lips, has been applied in patients with peripheral facial paresis and might also be useful in FSHD patients [88, 89]. For the Labbé technique to be successful, it must first be proven that the temporalis muscle is spared in disease progression. Muscle ultrasonography could be used to investigate this, as disease severity correlates with muscle ultrasound findings in FSHD patients [90].

*Cosmetic interventions.* FSHD patients at our expert centre have frequently asked for the applicability of botulinum toxins and dermal fillers for improving facial symmetry. Although we specifically added botulinum toxins in the search strategy, we did not find any study assessing this. Dermal fillers were used in one included case report. This case report by Dua et al. [66] describes the usage of dermal fillers and cog threads and results showed partial improvements. However, these results should be interpreted with caution, because of study design, lack of systematic outcome measurements, and limited follow-up duration (3 weeks). Botulinum toxins are used for improving synkinesis in facial palsy patients [91, 92]. But outcomes of studies focussing on synkinesis were not part of this review, because it is not an equivalent of facial weakness and therefore no recommendations on applicability to facial weakness can be made. Thus, not enough evidence is available to conclude whether botulinum toxins and dermal fillers are useful options in FSHD.

**Fig. 4:**
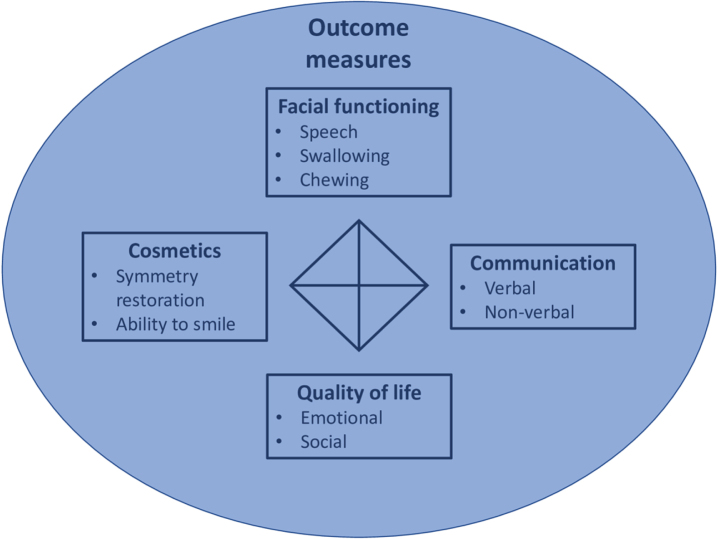
Different outcome assessment categories for assessing symptomatic treatments for improving altered facial expression. Four different outcome assessment categories: 1. facial functioning: consisting of speech, swallowing and chewing; 2. communication: consisting of verbal and non-verbal communication; 3. quality of life: consisting of emotional and social functioning; 4. cosmetics: consisting of symmetry restoration and ability to smile.

*Future studies.* Future studies on treatment options to improve altered facial expression should preferably address different categories of outcomes measures: cosmetic, facial functioning, communication, and quality of life (see Fig. 4). Not one of the included studies addressed all these categories. Psychosocial outcome measures were especially under-represented. In addition, to allow comparison between different studies, it would be recommended to use similar study designs and outcome measures.

### Limitations

Multiple neurological diseases were included, because of the expected scarcity of literature about treatment options for improving facial weakness in FSHD patients and of the expected wide applicability of certain symptomatic treatments. However, the anatomical location of the defect and the pathophysiology is different in these diseases (Fig. 2), therefore some recommended treatments are not applicable to FSHD.

When comparing the included diseases on altered facial expression, especially Parkinson’s disease has a different underlying mechanism. As bradykinesia causes the altered facial expression, whereas in the other included disease this is caused by facial weakness or facial paresis. Additionally, medication and associated on-off phenomenon can effect the degree of bradykinesia, potentially affecting the outcomes of symptomatic treatments. However, the included studies did provide valuable insights into current symptomatic therapies, which could be discussed for their potential applicability in FSHD.

Bell’s palsy patients have a high spontaneous recovery rate, as about seventy percent has a spontaneous recovery within three to six months [93]. Therefore, in studies with Bell’s palsy patients a high number of patients recovering after treatment, would also recover without treatment. Treatment effectivity could therefore be overestimated in this disease, this applies especially for studies performed in the acute phase of Bell’s palsy.

Of the included studies, 12 consisted of case reports. Level of evidence is low, and conclusions must be taken with caution. Nevertheless, these studies were included because they could point out treatment options that would otherwise not be identified and might be interesting for future research.

Studies performed before 1990 were excluded, because of the expected outdated treatment usage before 1990, especially for surgery techniques. Despite we could have missed important references.

## CONCLUSION

This systematic review showed that literature on treatments aiming to improve facial weakness in FSHD is scarce and has low level of evidence. Several potentially applicable treatment options for improving altered facial expression were identified after evaluation of the included therapies. These were: teaching (non-verbal) communication compensation strategies, speech training, physical therapy, and conference attendance. Smile restoration surgery could be an option for severely affected patients, but it is necessary to first identify facial muscles which are not or only mildly affected. Further research is necessary for all these potential treatment options. We recommend use of functional clinical outcome measures in various categories: cosmetic, facial functioning, communication, and quality of life. Ideally these outcome measures become standardized, to ensure future comparison between studies.

In short, this systematic review identified opportunities for future research, to achieve the goal in providing symptomatic treatment options for improving the consequences of facial weakness in FSHD.

## Data Availability

The data supporting the findings of this study are available within the article.
